# Primary closure of superior partial sternal cleft in a 2-month-old girl: case report

**DOI:** 10.1186/s43159-021-00113-8

**Published:** 2021-10-06

**Authors:** Halil Ibrahim Tanriverdi, Fulya Doğaneroğlu, Abdülkadir Genç, Ömer Yılmaz

**Affiliations:** Department of Pediatric Surgery, Manisa Celal Bayar University Medical School, Uncubozkoy, 45030, Yunusemre/Manisa, Turkey

**Keywords:** Sternal cleft, Primary closure, Hemangioma

## Abstract

**Background:**

Sternal cleft is a quite rare malformation. It is seen 1 out of 100,000 live births and makes up less than 1% of all chest wall deformities, seen more often among females. The deformity can be partial or complete. Partial deformities can be superior or inferior. It is generally diagnosed at birth when paradoxical respiratory movements are seen. Patients are often asymptomatic when they are born and generally other abnormalities accompany. As sternal clefts can be repaired primarily at early ages, they are repaired using autologous or synthetic grafts in the following years. We present a 2-month-old girl with superior partial sternal cleft repaired primary and accompanying hemangiomas in this case report.

**Case presentation:**

A girl who was born in another center and had a sternal cleft, who did not have any problems in the early period, was admitted to our hospital with respiratory distress at the age of 43 days. The patient was monitored with mechanical ventilator support, and there were hemangiomas around his left ear and lips. There were paradoxical respiratory movements in front of the heart, in the upper midline of the chest. Three-dimensional computed tomography showed that the upper part of the sternum did not develop, and there were hypoplasic sternal bars on both sides. It was evaluated as superior partial sternal cleft, and surgery was planned. In the operation, the sternal bars were released from the pericardium and pleura. The periosteum in the medial of both sternal bars was then released and connected in the midline, in front of the pericardium.

**Conclusion:**

Although neonates with a sternal cleft are asymptomatic at birth, respiratory symptoms may develop in later periods. In addition, because the structures are more flexible in the neonatal period, the primary repair of the cleft is easier and the risk of cardiac compression is lower. In our case, sternal bars could be approached primary, and no chondral grafts, patches, or steel wires were required.

## Background

Sternal cleft is a rare congenital anterior chest wall defect. It makes up less than 0.15% of all chest wall malformations and is seen more often in girls [1]. It is caused by the cessation of fusion of the sternal bars in the intrauterine period, between the 7th and 9th weeks. Although the cause is unknown, it is stated that it may be related to the Hox gene which provides the development of the embryo in the body plan along the front-back axis [2]. Partial and complete types exist. Partial deformities may be superior or inferior [3]. It is generally diagnosed at birth when paradoxical respiratory movements are seen. Patients are often asymptomatic when they are born. Sternal clefts usually isolated anomalies [4]. Sometimes, other anomalies may accompany. As sternal clefts can be repaired primarily at early ages, they are repaired using autologous or synthetic grafts in the following years. The 2-month old girl with primary repaired superior partial sternal cleft and accompanying hemangiomas is reported below. For the operation, we waited for the respiratory symptoms to get better and the pneumothorax to regress. The operation was performed on the 59th day of life.

## Case presentation

**Fig. 1:**
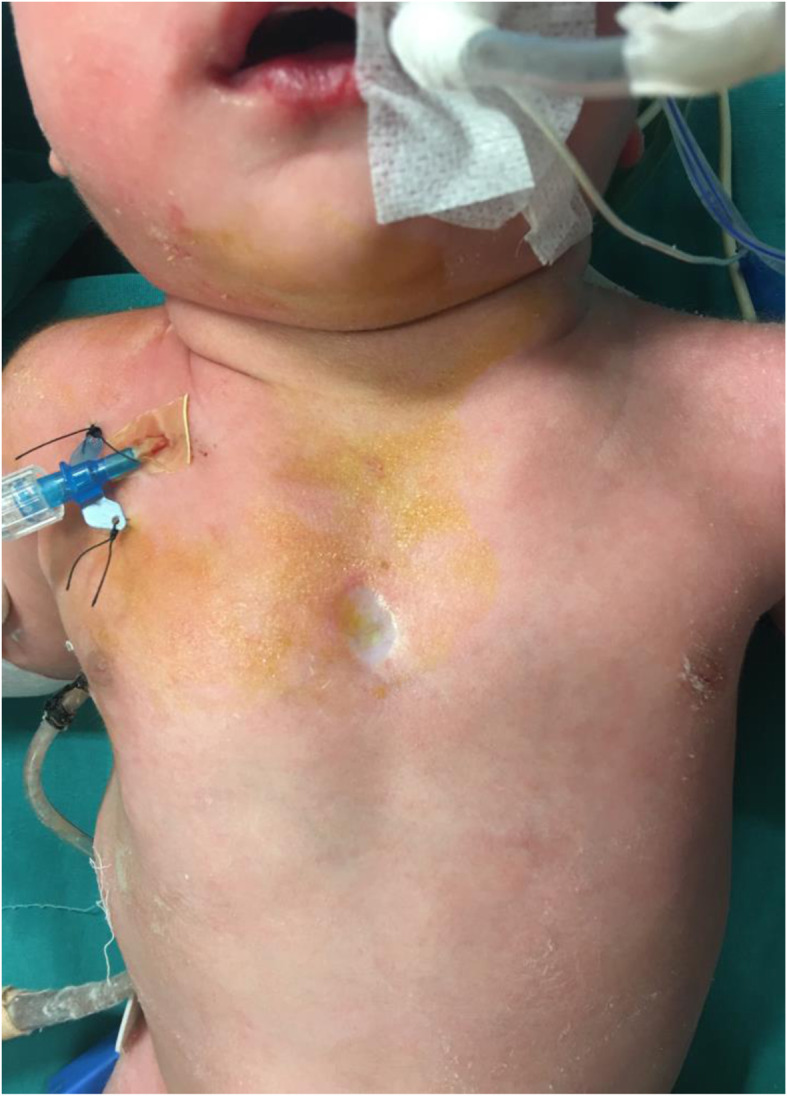
On the chest midline, the superior part of the skin which is in front of the heart was fibrotic, and paradoxical respiratory movements were present in this area

**Fig. 2:**
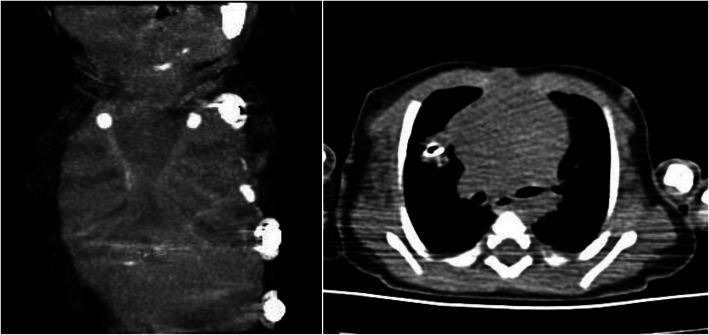
It is seen that the superior part of the sternum was undeveloped at CT. There were hypoplastic sternal bars bilaterally. Fusion defect of the superior sternum was present, and sternal bars united at the inferior

Our patient was born in another medical center with cesarean and is a full-term newborn aged 43 days and weighed 3180 g with a sternal cleft. She did not have any respiratory problems. In the early stage, an operation was not planned, and she was discharged from the other medical center. When the patient was 43 days old, she came to our emergency service with respiratory distress and wheezing. It was not prenatally diagnosed, and no additional features were found in family history. She was monitored in the intensive care unit and had hemangiomas around her left ear, around her lips, and in the oral mucosa. On the chest midline, the superior part of the skin which is in front of the heart was fibrotic, and paradoxical respiratory movements were present in this area (Fig. 1). The case was supported by noninvasive mechanical ventilation support due to respiratory distress, tachypnea, and intercostal and suprasternal retractions. The heart’s movements could be observed through the skin covering the defective area. No pathologies were detected in abdominal and transfontanelle ultrasonography along with normal lung X-ray and laboratory tests. Only the 1st to the 2nd grade tricuspid insufficiency was viewed in echocardiography. Because of increasing respiratory distress on the third day of monitoring, a lung X-ray was taken. Pneumothorax in the right hemithorax was seen in the X-ray, and tube thoracostomy was performed. On three-dimensional thoracic computed tomography (CT), it is seen that the superior part of the sternum was undeveloped (Fig. 2). There were hypoplastic sternal bars bilaterally. Fusion defect of the superior sternum was present, and sternal bars united at the inferior. The existing pathology was evaluated as superior partial sternal cleft, and operation was planned. No pathologies were found in the ophthalmological examination. The patient was intubated because respiratory distress was continuing. Cranial and abdominal magnetic resonance imaging (MRI) was normal. We waited for regression of respiratory symptoms and pneumothorax in order to operate on the patient. The operation was performed on the 59th day of her life.

**Fig. 3:**
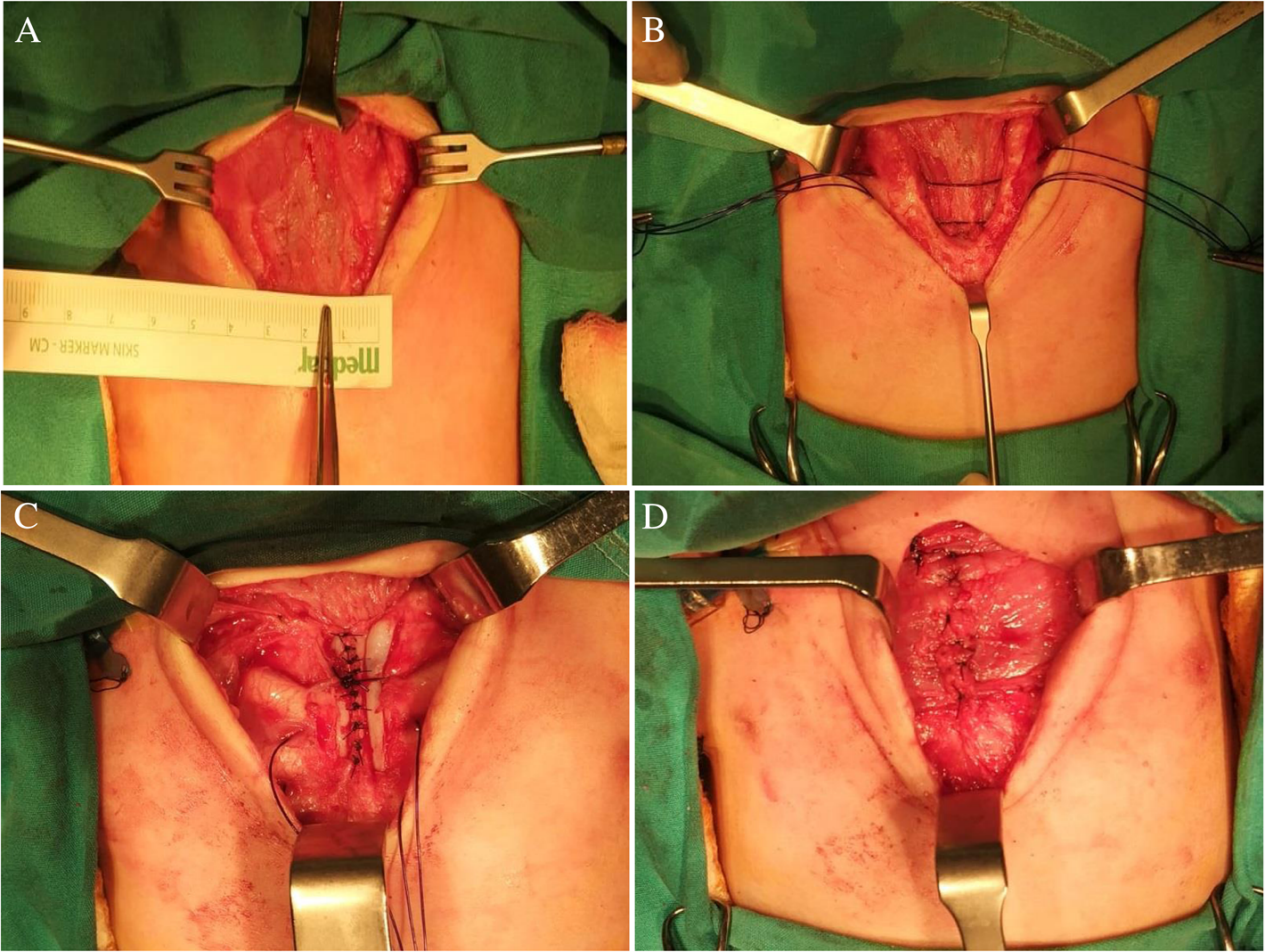
**A** There was a 3-cm opening at the widest part between the sternal bars. **B** U-shaped defective sternal bars were put in the middle by dissecting from the pleura and pericardium then sutures were placed behind the sternal bars. **C** The medial periosteum of both sternal bars was dissected and pulled together in front of the pericardium in the midline. **D** Sternal bars were connected to each other, and the defect was closed completely then the pectoral muscle flaps were put closer in the midline

**Fig. 4:**
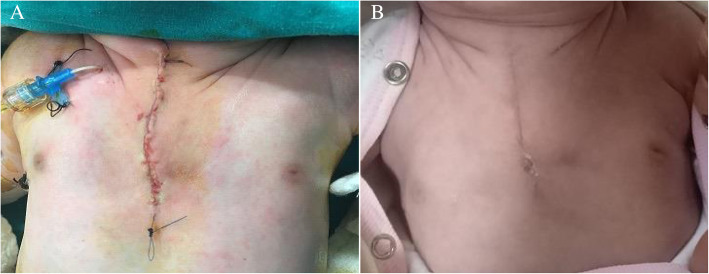
**A** Fibrotic skin tissue was excised, and the skin was closed. **B** The first year of the follow-up is uneventful, and sternal bars have not disconnected

Operation started with a median incision around the fibrotic skin patch in the chest midline. Subcutaneous tissues were dissected. There was a 3-cm opening at the widest part between the sternal bars (Fig. 3A). U-shaped defective sternal bars were put in the middle by dissecting from the pleura and pericardium. 2/0 polydioxanone sutures (PDS) were placed behind the sternal bars to be approximated later (Fig. 3B). Then, the muscle flaps were prepared by dissecting pectoral muscles bilaterally. Subsequently, the medial periosteum of both sternal bars was dissected and pull together in front of the pericardium in the midline (Fig. 3C). Then, the sternal bars were moved towards each other with 2/0 PDS. The patient’s hemodynamic and cardiac functions were monitored for 5 min. Due to the absence of any change in parameters, sternal bars were connected to each other, and the defect was closed completely. Earlier prepared pectoral muscle flaps were put closer in the midline in order to support the anterior chest wall (Fig. 3D). Fibrotic skin tissue was excised, and the skin was closed (Fig. 4A). The patient was monitored as curarized for 2 days postoperatively then was extubated on postoperative 4th day, transferred to service on postoperative 7th day, and finally was discharged on the 9th day. Currently, the patient is in the 18th month of postoperative follow-up. The patient has no problem, and sternal bars have not disconnected (Fig. 4B). She also received b-blocker therapy for her hemangiomas, and her hemangiomas regressed completely. Written consent to publish this information was obtained from study participants.

## Discussion

Sternal cleft is a quite rare entity. It is seen 1 out of 100,000 live births and makes up less than 1% of all chest wall deformities [5]. It is seen more often among females. The deformity may be partial or complete. Complete sternal cleft is rarer. Partial deformities can be superior or inferior [3]. Our case was female, and her cleft was partial located superiorly.

As sternal cleft can be diagnosed prenatally, it can be recognized after birth when abnormal heart movements are seen in the sternal region. In the beginning, patients are generally asymptomatic. There are also reported cases that were late-diagnosed [4, 6]. When sternal cleft is not diagnosed in the neonatal period and is not operated, gas exchange in the lungs is corrupted and respiratory dysfunction develops. These cause dyspnea, respiratory distress, chronic coughing, and recurrent respiratory system infections [4]. Our case was not diagnosed prenatally. She was evaluated in another medical center in the neonatal period and was discharged without any surgical intervention due to the absence of symptoms. The case who was not operated on in the early period came to our hospital with respiratory distress when she was 1.5 months old.

Three-dimensional CT imaging is useful in the assessment of sternal cleft’s type, location, size, and relation with the heart and main blood vessels. Planning of the operation must be done accordingly. Additionally, cardiac functions and abnormalities must be evaluated with echocardiography. Also, cardiac functions must be evaluated in terms of a possible compression, after the operation.

Sternal clefts are usually isolated anomalies, but sometimes other anomalies may accompany [4]. While facial hemangiomas accompany with a superior partial cleft, inferior partial cleft may be seen with ectopia cordis and Cantrell Pentalogy [4]. The coexistence of sternal cleft and vascular lesions was defined by Hersh for the first time [7]. It is shown that PHACE syndrome (posterior fossa brain abnormalities, hemangiomas, cranial vascular abnormalities, aortic coarctation, cardiac defects, eye abnormalities) and sternal malformations are seen together [8]. Sternal malformations, vascular dysplasia, and PHACE syndrome are thought to be of the common origin [9]. PHACE syndrome is defined as PHACES syndrome with the addition of sternal cleft [4]. Other than those, pectus excavatum may be also seen with a sternal cleft. In our case, hemangiomas accompanied with a superior partial sternal cleft. There were hemangiomas around the left ear, around the lips, and in the oral mucosa. Cranial MRI and echocardiography were normal. There were not any cranial or cardiac abnormalities. No additional anomalies were seen in the thorax, and ophthalmologic examination was normal. The hemangiomas were not wide, so they were treated with medical therapy and they regressed quickly.

Cleft repair is done for both the maintenance of respiratory hemodynamic and protection of the heart and main blood vessels. Also, it might be done for cosmetic reasons [6]. Sternal cleft can be repaired primary or autologous and synthetic grafts with different techniques according to operation age. Bone graft interposition, muscle flap interposition, or synthetic patches can be used [4, 6, 10–13]. Sabiston described sliding chondrotomies which oblique incisions through the cartilages for increasing their length [14]. Meissner divided the costal cartilages laterally and rotated them medially to close the defect [15]. Autologous grafts such as costal cartilage, rib, cranial bone, and tibia were used to close the defect [16, 17]. Also, prosthetic materials such mesh, Teflon, silicone, acrylic, and titanium were used for closing the defect [16, 18, 19]. Especially for older patients, synthetic materials may be needed because of the loss of elasticity. However, infection and fluid collection might cause problems in these cases. Generally, primary repair can be done in cases younger than 3 months [20]. Because the tissues are more flexible, open edges get closer easily, and cardiac compression risk lowers when sternal cleft is repaired in the early period [1, 4]. Some authors recommend operating these patients on the first a few weeks after birth [1, 4, 6, 11]. Because the structures are more flexible in the neonatal period, the primary repair of the cleft is easier and the risk of cardiac compression is lower. In our case, sternal bars could be approached primarily, and no chondral grafts, patches, or steel wires were required. Because our case was 2 months old, the cleft could be primarily repaired. No evidence of cardiac compression appeared during and after the operation.

Because our case was asymptomatic, an early operation was not planned in the hospital where she was initially evaluated. However, the patient applied to the emergency service with respiratory distress and mechanical ventilation was required. Meanwhile, pneumothorax developed, and we waited for respiratory symptoms to get better and pneumothorax to regress in order to be able to perform the operation. However, the patient could be operated on in a more convenient and comfortable way, in her first month after birth. Fortunately, the defect in our patient could be primarily repaired, and no problems occurred in the postoperative period.

## Conclusion

Although neonates with a sternal cleft are asymptomatic at birth, respiratory symptoms may develop in later periods. Neonates who are diagnosed with a sternal cleft should be followed closely, even maybe operated on without discharge and respiratory distress, and should not be expected to grow.

## Data Availability

All data and materials are available in hospital records.

## Abbreviations

CT: Computed tomography
MRI: Magnetic resonance imaging
PDS: Polydioxanone suture
